# Morphological, Histo-Morphometric and Histochemical Studies on Compartment 2 of Dromedary Camel (*Camelus dromedarius*) Stomach

**DOI:** 10.3390/vetsci13070630

**Published:** 2026-06-29

**Authors:** Zarroug Hassan Ibrahim

**Affiliations:** 1 Department of Medical Biosciences, College of Veterinary Medicine, Qassim University, Buraydah 52571, Saudi Arabia; z.ibrahim@qu.edu.sa; 2 Department of Biomedical Sciences, College of Veterinary Medicine, Sudan University of Science and Technology, Khartoum 11111, Sudan

**Keywords:** camel stomach, compartment 2, morphology, gross anatomy, histology, histochemistry

## Abstract

The second stomach compartment (C2) of the dromedary camel (*Camelus dromedarius*) plays an important role in digestion. However, detailed morphological and histochemical data remain limited. This study aimed to investigate the gross anatomy, histological organization, histometric features, and histochemical distribution of muco-substances in C2. The study was conducted on twenty dromedary camels, including fetuses and adults. Gross anatomical observations were performed on eight fresh and fixed specimens, while histological, histometric, and histochemical analyses were carried out on samples from twelve adult camels using routine and special staining techniques to identify neutral and acidic mucins. C2 was the smallest gastric compartment, located on the right side of the abdominal cavity and partially continuous with C1. Its mucosa formed chambered zones supported by prominent longitudinal muscular bands. Histologically, C2 comprised glandular and non-glandular regions. The glandular mucosa contained gastric pits and branched tubular glands with mucous, chief, and parietal cells, whereas the non-glandular region was lined by keratinized stratified squamous epithelium. Submucosal lymphoid aggregations were observed near the C2–C3 junction. Histometric analysis revealed a markedly developed tunica muscularis. Strong PAS and Alcian blue reactions indicated abundant neutral and acidic mucins. These findings demonstrate that C2 is a structurally specialized compartment supporting digestion, mucosal immune defense, and adaptation to arid environments, clearly distinguishing it from the reticulum of true ruminants.

## Simple Summary

The second stomach compartment (C2) of the dromedary camel is important for digestion, yet its structure and secretory features are poorly understood. This study describes the gross anatomy, microscopic structure, layer thickness, and mucin composition of C2 in fetal and adult camels. C2 was the smallest compartment, with distinct glandular and non-glandular regions, strong muscular development, and abundant neutral and acidic mucins. The presence of submucosal lymphoid tissue reflects immune defense and, together with mucin secretion, keratinization, and strong musculature, indicates adaptation to fibrous digestion and harsh desert conditions.

## 1. Introduction

The dromedary camel (*Camelus dromedarius*) is considered a pseudo-ruminant because it possesses a three-compartment stomach composed of compartment 1 (C1), compartment 2 (C2), and compartment 3 (C3), in contrast to true ruminants that have a four-compartment stomach [1,2,3,4]. However, some authors have described four gastric compartments in the dromedary camel, separating the cranial tubular portion of the third compartment as C3 and the caudal dilated portion as a distinct compartment (C4) [5,6]. Functionally, early investigations of the camelid stomach revealed certain structural similarities to the stomach of true ruminants, while also highlighting distinct features related to desert adaptation [4,7,8].

In true ruminants, the reticulum—considered functionally equivalent to C2 in camelids—has been extensively studied with respect to morphology and histochemistry due to its essential roles in ruminal motility, fermentation, and regurgitation [9,10,11,12]. The mucosal architecture of the bovine reticulum contributes directly to rumination efficiency, mixing of forestomach contents, and the transfer of food particles to the omasum [13]. In sheep and goats, the absorptive capacity of volatile fatty acids is influenced by epithelial keratinization and muco-substance distribution, which vary with diet type and age [9,11].

Histochemical studies have demonstrated that the reticular epithelium of true ruminants secretes both neutral and acidic mucopolysaccharides, reflecting a combination of absorption and protective functions [12]. In camelids, however, distinct differences have been documented. The presence of both acidic glycoconjugates and neutral polysaccharides within the glandular sacs of the Bactrian camel has been reported, suggesting important roles in lubrication, epithelial protection, and absorption [7]. Among the limited investigations focusing on C2 of the dromedary camel, Al-Aiyan et al. [14] described age-related morphological variations in the mucosal and muscular layers, emphasizing structural specializations that support fermentation, absorption, and mechanical processing of ingesta.

Despite its presumed functional importance, morphological and histological studies of the C2 segment of the dromedary camel stomach remain limited compared to other stomach segments. Previous studies have mainly addressed the general morphology and histochemistry of the camel stomach, particularly segments C1 and C3, including glandular structure, myosin composition, and age-related changes [2,4]. Al-Ayan et al. [14] provided valuable histological data and histological measurements of segment C2, describing the differences in mucosal and muscular layers and confirming its role in mechanical processing and digestion. However, many aspects remain insufficiently described, including the detailed regional organization of glandular and non-glandular areas, comprehensive quantitative comparisons of tissue layers, and the distribution and importance of neutral and acidic mucins in relation to digestive adaptation. Consequently, the structural and functional specializations of the C2 segment are less clear than those of similar segments in true ruminants.

Therefore, this study investigates the gross anatomy, histological organization, histometric characteristics, and histochemical localization of muco-substances in C2 of the dromedary camel. By integrating these approaches, the study seeks to clarify the tissue organization, secretory activity, and adaptive functional roles of C2, thereby filling existing gaps and contributing to a more comprehensive understanding of camelid digestive physiology.

## 2. Materials and Methods

### 2.1. Animals

The camels included in this study were slaughtered for human consumption at Buraydah Central Slaughterhouse, Saudi Arabia, in accordance with the regulations of the Saudi Ministry of Environment, Water, and Agriculture. No animals were euthanized or subjected to experimental procedures specifically, and tissue samples were collected post-mortem. Therefore, ethical approval was not required for this study.

### 2.2. Gross Anatomical Study

For the gross anatomical investigation, eight camels (five adults and three fetuses) were examined. One fetus was studied unfixed, while the remaining two fetuses and the stomachs of all adult animals were fixed in 10% formalin for one week. After fixation, the specimens were carefully dissected to demonstrate the shape, location, anatomical relationships, and internal surface of compartment 2 (C2).

### 2.3. Microscopic Study

**Figure 1:**
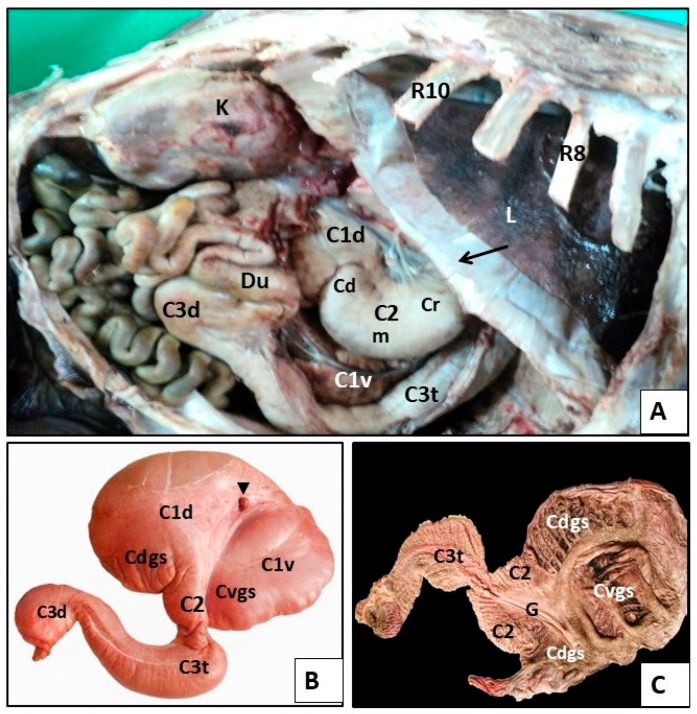
(**A**) Right lateral view of the abdominal cavity of a camel fetus after removal of the liver. (**B**) Right lateral view of the dromedary camel stomach complex. (**C**) Internal view of the dromedary camel stomach complex. Shown are compartment 2 (C2) with its cranial (Cr), middle (m) and caudal (Cd) regions, the eighth (R8) and tenth (R10) ribs, diaphragm (Arrow), right lung (L), right kidney (K), duodenum (Du), caudo-dorsal (C1d) and cranio-ventral (C1v) sacs of compartment 1, tubular (C3t) and distended (C3d) parts of compartment 3, esophagus (arrowhead), esophageal groove (G), and the caudo- dorsal (Cdgs) and cranio-ventral (CVgs) glandular sacs.

The microscopic study was performed on C2 samples collected from twelve adult camels immediately after being slaughtered. From each compartment, tissue samples were collected from the cranial, middle, and caudal regions (Figure 1A), with three samples collected per region. The samples were fixed in 10% formalin and then routinely processed, and paraffin sections were prepared as earlier described [15]. The histological evaluation was conducted on paraffin sections of six animals stained with hematoxylin and eosin (H&E) for general histological structure. Masson’s trichrome was used for smooth muscle and collagenous fibres and Gordon & Sweet’s for reticular fibres [15]. The morphometric study was performed on the same H&E-stained sections used in the histological study (six camels) for measurements of the thickness of the mucosa, submucosa, muscularis, and serosa of the glandular and non-glandular regions of C2. Measurements were performed under a Leica microscope (DMD108, Wetzlar, Germany) and the mean values from the various sample sites were calculated for each layer, and differences were considered significant at *p* < 0.05 using Student’s *t*-test.

The histochemical investigation was conducted on paraffin sections obtained from the remaining six animals using periodic acid–Schiff (PAS) staining to detect neutral muco-substances, Alcian blue (pH 2.5) staining, counterstained with nuclear fast red, to demonstrate acidic muco-substances, and a combined Alcian blue (pH 2.5)/PAS staining sequence for mucin differentiation.

During the preparation of this manuscript the author used Microsoft Copilot (Microsoft 365 Copilot), accessed in May 2026, for assistance in preparation of the graphical abstract and in refining the interpretation of some results.

## 3. Results

### 3.1. Gross Anatomical Observations

Compartment 2 (C2) was the smallest of the three gastric compartments of the dromedary camel. It was oval- to bean-shaped and located on the right side of the abdominal cavity, extending between the eighth and tenth ribs (Figure 1A,B). Externally, C2 was separated from the cranioventral and caudo-dorsal sacs of C1 by distinct cranial and caudal grooves, respectively. Internally, the cranial and caudal portions of C2 were closely associated with the cranioventral and caudo-dorsal glandular sacs of C1.

C2 communicated with C3 through a narrow orifice, while remaining incompletely separated from the cranioventral sac of C1, indicating partial anatomical continuity between these compartments (Figure 1C).

**Figure 2:**
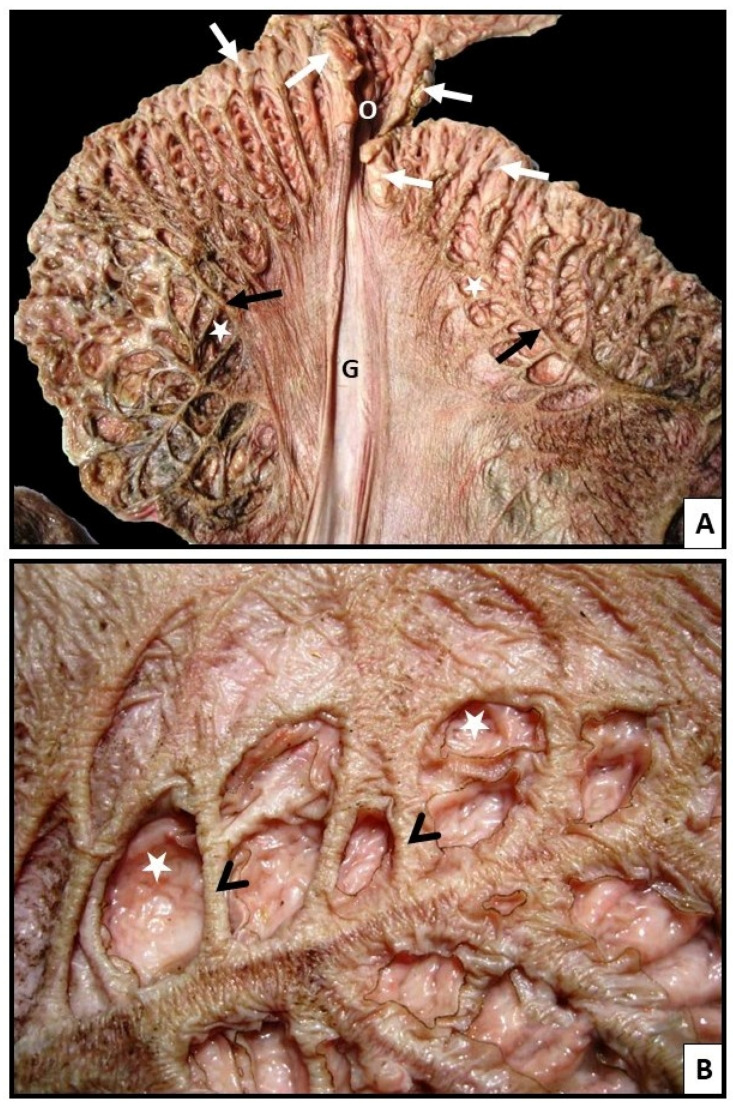
(**A**,**B**) Internal view of compartment 2 of the adult dromedary camel stomach showing the esophageal groove (G), large muscular bands (black arrows), small muscular bands (arrowheads), and small chambers (stars). Note the irregular lymphatic folds (white arrows) surrounding the opening (O) leading to compartment 3.

The internal surface of C2 exhibited prominent, parallel longitudinal muscular large bands that branched into smaller interconnected bands, forming numerous chambers of varying shapes and sizes (Figure 2A,B). The esophageal groove originated at the gastro-esophageal opening on the dorsal aspect of C1 and extended along the craniodorsal internal surface of C1 and C2 before opening into C3 (Figure 2A). The groove displayed a smooth interior surface devoid of muscular bands and chamber formation.

### 3.2. Histological Observations

**Figure 3:**
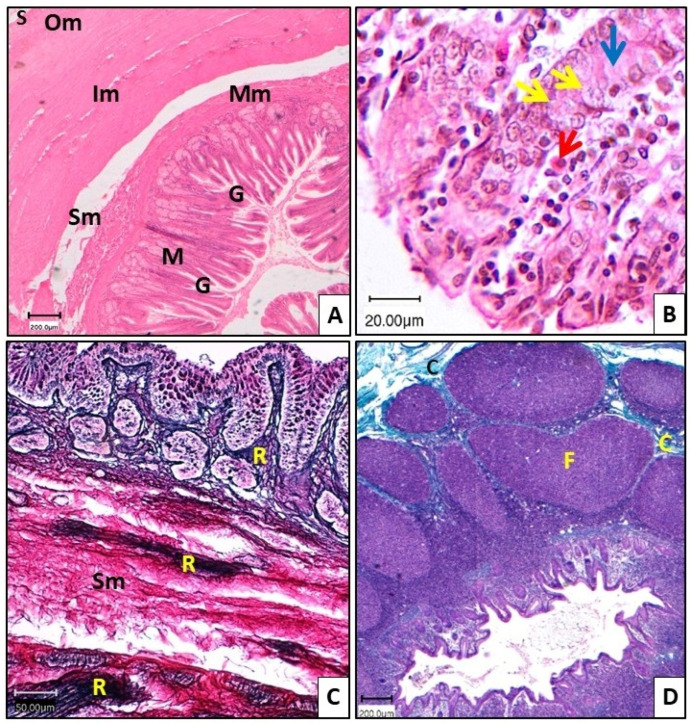
Glandular region of the second stomach compartment (C2) of the dromedary camel. (**A**,**B**) H&E sections showing mucosa (M) with branched tubular glands (G), muscularis mucosa (Mm), submucosa (Sm), muscularis (inner circular, Im; outer longitudinal, Om), and serosa (S). Chief (blue), parietal (red), and mucous cells (yellow arrows) are indicated. (**C**) Gordon and Sweet’s stain showing reticular fibers (R). (**D**) Masson’s trichrome stain showing collagen fibers (C) and lymphoid follicles (F). Scale bars: (**A**,**D**) = 200 µm; (**B**) = 20 µm; (**C**) = 50 µm.

**Figure 4:**
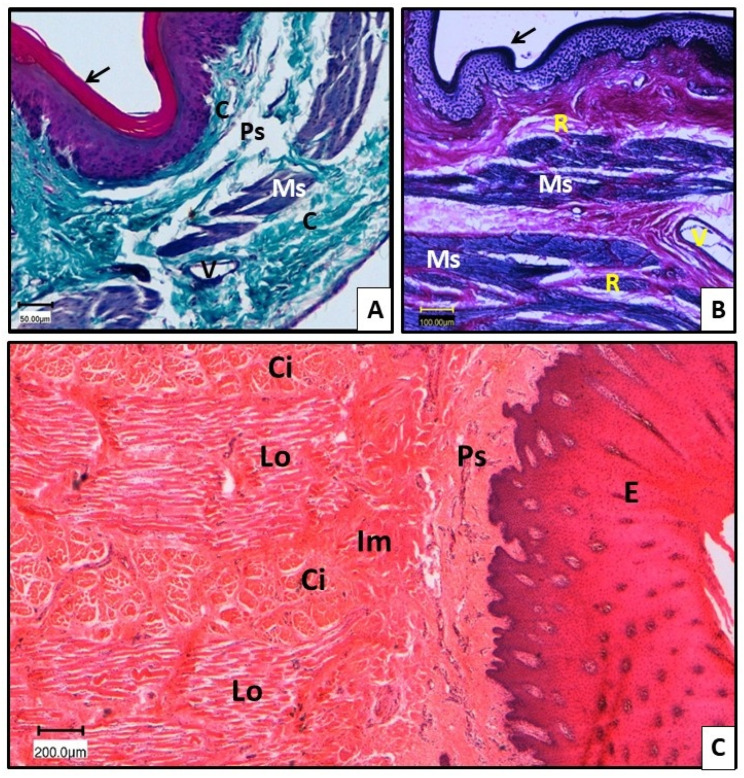
Non-glandular region of the second stomach compartment (C2) of the dromedary camel showing (**A**) stratified squamous epithelium with a thin keratin layer (arrows) and collagen fibers (C) in the propria-submucosa (Ps) (Masson’s trichrome), (**B**) reticular fibers (R) and blood vessels (V) within the propria-submucosa and between muscle layers (Ms) (Gordon and Sweet’s), and (**C**) thick keratinized stratified squamous epithelium (E), propria-submucosa (Ps), inner circular muscle (Im), and a thick outer muscular layer composed of longitudinal (Lo) and circular (Ci) bands (H&E). Scale bars: (**A**) = 50 µm; (**B**) = 100 µm; (**C**) = 200 µm.

The interior of C2 showed both glandular and non-glandular regions. While the glandular mucosa covered the floors of the chambers, the non-glandular mucosa covered the large and small muscular bands as well as the esophageal groove and surrounding area. There were four histological layers forming the wall of both regions: tunica mucosa, tunica submucosa, tunica muscularis, and tunica serosa (Figure 3 and Figure 4).

### 3.3. Glandular Region

The tunica mucosa in the glandular region was folded and had gastric pits and was lined by one layer of columnar epithelium (Figure 3A,B). The lamina propria consisted mainly of tubular and branched simple glands that opened into the pits. Loose connective tissue containing blood vessels, lymphatics and collagenous and reticular fibers were also present (Figure 3B,C). Three types of cells were observed in the deeper part of the pits and the glands which were neck cells, chief cells and parietal cells. The neck cells were mucous with basal nuclei, the chief cells were basal and cuboidal in shape and had basophilic cytoplasm and dark round nuclei and the parietal cells were larger than chief cells and had eosinophilic cytoplasm and large nucleus (Figure 3B). The thin muscularis mucosa consisted mainly of smooth muscle fibres which separated the lamina propria and submucosa.

The submucosa, which was a relatively thick layer, was made up of loose connective tissue rich in blood vessels and contained collagenous fibres and reticular fibres (Figure 3D). Lymphoid aggregation in the form of diffuse lymphatic tissue and lymphatic follicles was observed in the submucosa of compartment 2 sections collected from the area around the opening between compartment 2 and compartment 3 (Figure 3D). The lymphatic tissue in most sections extended towards the lamina propria and protruded towards the lumen.

The tunica muscularis contained two layers of smooth muscle fibres: an inner circular layer and an outer longitudinal layer. Blood vessels and fat cells were observed among the muscle fibres of each layer and between the two layers.

The serosa was a thin layer of loose connective tissue (sub-mesothelium) covered by a single layer of squamous cells (mesothelial layer).

### 3.4. Non-Glandular Region

The mucosa of the non-glandular region was lined by a keratinized stratified squamous epithelium which was extremely thicker and less folded in large longitudinal bands than in smaller interconnected ones (Figure 4). The lamina propria and submucosa were merged as one layer (propria-submucosa) as the muscularis mucosa was absent. It consisted of loose connective tissue with scattered reticular and collagenous fibers, smooth muscle fibers, and blood vessels. In the interconnected bands it had two parts: an outer papillary part with loose connective tissue, and an inner thick reticular part which was mainly formed of collagenous and reticular fibers with some smooth muscle fibres, blood vessels and adipose tissue (Figure 4A,B).

The tunica muscularis was the thickest layer and it formed the support of the small and large bands. It consisted of an inner circular and outer smooth muscle layer with some blood vessels and nerve fibres in between. The outer layer of the large bands showed a thick longitudinal muscular layer which formed interconnected longitudinal and circular muscular bands (Figure 4C).

The tunica serosa in the non-glandular region consisted of mesothelium and sub-mesothelium.

### 3.5. Histometry

Significant variations in tissue layer thickness were observed within each band type, as well as between symmetrical layers in small and large bands (Table 1). In small bands, the outer longitudinal muscular layer was the thickest, followed by the inner circular muscular layer, while the epithelial layer was the thinnest. The propria-submucosa and serosa showed intermediate values. Similarly, the outer longitudinal muscular layer of the large bands recorded the greatest thickness, followed by the epithelial layer and then the inner circular muscular layer, while the propria-submucosa and serosa were markedly thinner. Across all corresponding layers, the large bands were significantly thicker than the smaller ones.

In the glandular region, there was a significant difference in thickness between the histological layers of compartment 2 (Table 2). The outer longitudinal muscle layer was the thickest, followed by the inner circular muscle layer, while the tunica mucosa represented the thinnest layer. All histological layers were distinct except for the serosa and propria-submucosa, which did not show any significant difference.

### 3.6. Histochemistry

**Figure 5:**
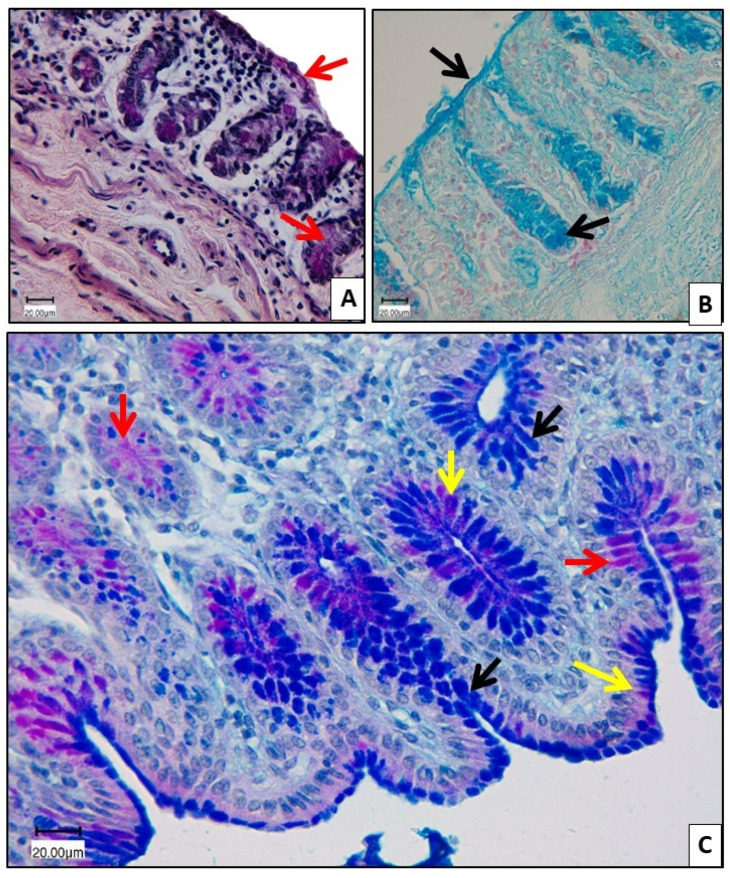
Histochemical reactions in the glandular region of the second stomach compartment (C2) of the dromedary camel showing (**A**) PAS-positive reactions and (**B**) Alcian blue (pH 2.5)-positive reactions in the surface epithelium and basal glandular cells (arrows), and (**C**) combined PAS/Alcian blue staining demonstrating strong PAS-positive (red arrows), Alcian blue-positive (black arrows), and mixed PAS/Alcian blue reactions (yellow arrows) in epithelial and glandular cells.

Periodic Acid–Schiff (PAS) reaction (Figure 5A) revealed a strongly positive staining in the epithelial cells, especially in their apical cytoplasm. The glandular cells also showed strong PAS-positive reactions in glandular and surface epithelial cells, especially in their apical parts. On the other hand, the smooth muscles, blood vessels and connective tissue of lamina propria and submucosa showed a weak to moderate PAS reactivity.

AB (pH 2.5) staining (Figure 5B) was strong in the surface part of epithelial cells and the basal glandular cells. Moderate reaction was observed in the gastric pits and mucous neck cells. AB (pH 2.5) reaction in the mucosal smooth muscle and connective tissue of the lamina propria showed minimal affinity.

The PAS/Alcian blue (pH 2.5) sequence (Figure 5C) showed three distinct colors which were blue (AB-positive), red (PAS-positive), and purple (AB and PAS-positive). A strong PAS-positive staining was shown in the surface epithelial and glandular cells. Strong alcian blue pH 2.5 positive reaction was also evident in numerous surface epithelial and glandular cells. Some epithelial and glandular cells exhibited a mixed reaction (purple color), reflecting the coexistence of AB and PAS reactions in these cells (Figure 5C). This mixed reaction was more noticeable within the glandular epithelial cells and deeper pit regions, whereas the surface epithelial cells predominantly displayed PAS-positive reaction. The connective tissue of the lamina propria showed weak or negligible staining with both PAS and Alcian blue.

## 4. Discussion

Although some authors [5,6] have proposed that the stomach of the dromedary camel comprises four compartments (C1–C4), based on subdivision of the third compartment into a cranial tubular part (C3) and a caudal dilated part (C4), the findings of the present study do not support this interpretation. In the examined specimens, these two regions of C3 appeared as continuous anatomical structures without distinct boundaries in their wall organization. These observations are consistent with the classical interpretation of the camel stomach as a three-compartment system (C1, C2, and C3) [1,2,3,4]. The absence of clear structural demarcation suggests that the tubular and distal portions of C3 represent functional variations within a single compartment rather than separate anatomical units, despite previous morphological descriptions [5,6].

The present anatomical findings showed that the second stomach compartment (C2) is the smallest gastric compartment in the dromedary camel and is located on the right side of the abdomen, extending between the eighth and tenth ribs. These observations agree with previous descriptions in camelid species, where C2 has been reported as an oval- or bean-shaped chamber situated between C1 and C3 and closely related to the liver [2,4,16]. Unlike true ruminants, which exhibit a clear anatomical separation between the rumen and reticulum, the dromedary camel stomach shows partial intercommunication between compartments, particularly between C1 and C2 [16]. In the present study, the presence of a deep groove partially separating C2 from the cranio-ventral sac of C1 supports the view that C2 represents a specialized extension of C1 rather than an independent compartment. This partial connection may facilitate coordinated motility and regulation of ingesta transfer between compartments. The relationship between C1 and C2 in the camel stomach remains controversial. Some studies suggest that C2 represents a specialized continuation of C1, based on their similar morphological and functional characteristics, including the presence of glandular and non-glandular mucosa and their shared roles in fermentation and absorption [14,17,18]. In contrast, other classical anatomical and physiological studies regard C2 as a distinct compartment comparable to the reticulum of true ruminants due to its structural organization, the presence of a defined connecting orifice with C1, and its involvement in digesta sorting and motility [19,20].

The internal architecture of C2 was characterized by parallel longitudinal muscle bundles that branched into smaller interconnected bands, forming numerous chambers of variable shapes and sizes. This arrangement suggests a specialized mechanical design that enhances flexibility in mixing, sorting, and redistributing ingesta. Similar compartmentalized musculature has been described in camelids [1,2]. In contrast, the reticulum of true ruminants exhibits a less complex muscular organization, reflecting functional differences in ingesta handling [1,13].

In the present study, a well-developed esophageal groove extended from the gastro-esophageal orifice through C1 and toward C3, representing an important adaptive feature. While the esophageal groove in ruminants is especially important for the calf during nursing, it remains structurally prominent in adult camels, serving as a direct pathway allowing fluids to bypass fermentation chambers [2,16]. The smooth inner surface of the groove, devoid of muscular bands or chambers, supports efficient fluid transport to C3 and contributes to water conservation and selective routing of ingesta—key adaptations to arid environments.

The current observations confirmed the coexistence of glandular and non-glandular mucosa within C2, accompanied by marked muscular development. The glandular mucosa was restricted to the floors of the chambers, whereas the muscular bands and esophageal groove were lined by non-glandular stratified squamous epithelium, as previously reported in camels [2,14,16]. This regional differentiation indicates functional specialization, with mechanically stressed areas protected by keratinized epithelium and chamber floors providing localized secretory activity.

Histological examination of the glandular regions revealed gastric pits lined by simple columnar epithelium and well-developed tubular and branched glands composed of mucous, chief, and parietal cells, indicating active gastric secretion. Similar glandular characteristics have been described in C2 and C3 of camels, distinguishing them from the completely non-glandular forestomach of true ruminants [1,4,21]. The structural similarity of these glands to typical gastric glands suggests roles in acid, enzyme, and mucus secretion, contributing to chemical digestion and regulation of luminal contents.

Prominent lymphoid aggregations were consistently observed within the submucosa of C2, particularly near the C2–C3 junction. These appeared as diffuse lymphoid tissue and lymph nodes extending toward the lamina propria, as also described by [22]. This finding suggests an active role in mucosal immune surveillance at a critical transition zone exposed to continuously changing physical, chemical, and microbial conditions. In camelids, digestion is characterized by prolonged retention and fermentation of fibrous, low-quality forage, which increases exposure to diverse microbial populations. Under harsh desert conditions, marked by dehydration, irregular feeding patterns, and the ingestion of dry, coarse, and often contaminated plant material, this exposure is further intensified. The presence of well-developed lymphoid tissue in C2 of dromedary camel was therefore suggested to reflect an adaptive morphological specialization that enhances mucosal protection, limits microbial translocation, and maintains epithelial integrity [22,23]. This immune component acts in concert with other structural features of C2, including mucin-rich secretion, keratinized epithelium, and strong musculature, collectively supporting digestive efficiency and resilience to environmental stressors characteristic of camelid habitats.

The non-glandular region of C2 exhibited a thick keratinized stratified squamous epithelium, particularly within the large longitudinal muscular bands. This, along with the absence of the muscularis mucosa and the fusion of the lamina propria and submucosa, reflects adaptation to mechanical stress and differs from the more uniform epithelial organization of the ruminant reticulum [1,2]. The increased epithelial thickness likely provides protection against friction and compression during vigorous mixing and contraction.

A distinctive feature of C2 was the strong development of the tunica muscularis, especially the thick outer longitudinal layer forming interconnected longitudinal and circular bands. This muscular specialization supports the interpretation that C2 functions primarily as a motility-oriented compartment involved in mixing, sorting, and propulsion of ingesta. Similar conclusions were reported in previous morphometric studies, which demonstrated the predominance of muscular layers over mucosa in both regions of C2 [14]. Although the reticulum of true ruminants also contributes to mechanical processing, its lack of mucosal glands and different muscular organization further emphasize that C2 is functionally analogous but not homologous to the ruminant reticulum [1,16].

Histometric analysis revealed clear regional and layer-specific specialization, with the outer longitudinal muscle layer significantly thicker in large bands than in smaller ones. Comparable observations were reported by [14], highlighting the functional importance of tunica muscularis variability. The dominance of muscular layers over tunica mucosa underscores the primary mechanical role of C2, while the coexistence of glandular and non-glandular mucosa indicates functional segmentation unique to camelids and distinct from true ruminants [1,2].

Histochemical findings demonstrated strong PAS positivity in surface and glandular epithelial cells, indicating abundant neutral mucins, while intense Alcian blue (pH 2.5) reactivity reflected a high content of acidic mucins, particularly sialomucins. Mixed PAS–Alcian blue reactions indicated co-expression of neutral and acidic mucins within individual cells. Similar patterns have been reported in camelids, although the intensity of acidic mucin expression appears more pronounced in dromedaries [2,4,16,24]. Acidic mucins enhance mucus viscosity, water retention, and resistance to microbial and mechanical stress, contributing to epithelial protection under harsh desert conditions [25]. In contrast, true ruminants predominantly exhibit PAS-positive neutral mucins in glandular regions, with acidic mucins being more restricted [26,27]. The observed mucin heterogeneity in C2 likely supports both lubrication and mucosal defense, reflecting an adaptive strategy for coping with environmental and dietary challenges.

The present study highlights key findings that further elucidate the structural specialization of C2 in dromedary camels, particularly its muscular organization, glandular mucosa, and immune architecture. The markedly developed outer longitudinal muscle layer, arranged into interconnected bands, represents a distinctive feature that facilitates localized mixing and redistribution of ingesta. This muscular dominance, supported by histometric analysis, exceeds previous reports and contrasts with the less specialized ruminant reticulum, indicating adaptation to fibrous diets. Histochemical analysis demonstrated a clear coexistence of neutral and acidic mucins within epithelial and glandular cells, as shown by strong PAS and Alcian blue reactions and their combined expression. This dual mucin system likely enhances lubrication, epithelial protection, and resistance to microbial and mechanical stress, appearing more pronounced than previously described in camelids. In addition, well-developed submucosal lymphoid aggregations, particularly near the C2–C3 junction, indicate a strategic immune component at a transitional zone exposed to variable ingesta and microbial challenges. Their extension toward the lamina propria supports a role in mucosal immune surveillance. Collectively, these findings demonstrate that C2 is a highly specialized organ integrating mechanical, secretory, and immune functions, reflecting adaptation to the environmental and dietary conditions of camelids.

## 5. Conclusions

It can be concluded that compartment 2 of the dromedary camel stomach is a structurally specialized organ characterized by strong musculature, mixed mucosal types, diverse mucin secretion, submucosal lymphoid tissue, and well-developed glandular com-ponents. These features support not only mechanical digestion, epithelial protection, and immune surveillance, but also active chemical digestion through acid, enzyme, and mucus secretion. This multifunctional organization reflects adaptation to harsh desert conditions and clearly distinguishes C2 from the reticulum of true ruminants.

## Figures and Tables

**Table 1:** The mean thickness (μm) of the layers of the non-glandular region of the second compartment of dromedary camel stomach.

	Epithelium	Propria-Submucosa	Inner Muscle Layer	Outer Muscle Layer	Serosa
Small bands	101.58 ± 6.63 ^a^	162.23 ± 24.69 ^b^	412.38 ± 19.30 ^c^	1092.43 ± 96.32 ^d^	287.08 ± 32.89 ^e^
Large bands	1558.83 ± 119.85 ^e^	373.17 ± 23.59 ^d^	887.25 ± 94.29 ^b^	2703.83 ± 89.55 ^c^	387. 08 ± 32.98 ^a^

Data are presented as mean ± SD. Means sharing different letters in the same row or the same column indicate statistically significant differences.

**Table 2:** The mean thickness (μm) of the layers of the glandular region of the second compartment of dromedary camel stomach.

	Mucosa	Submucosa	Inner Muscle Layer	Outer Muscle Layer	Serosa
Thickness	182.62 ± 16.98 ^ab^	235.87 ± 5.04 ^bc^	422.80 ± 20.30 ^d^	1131.83 ± 80.20 ^e^	265.00 ± 46.99 ^af^

Values are shown as mean ± SD. Means bearing different letters are significantly different.

## Data Availability

The data presented in this study are available within the manuscript.
